# Dietary modulation of intestinal integrity and functionality in weaned piglets using short-chain fructooligosaccharides, essential oils and sodium humate

**DOI:** 10.3389/fvets.2026.1771820

**Published:** 2026-03-04

**Authors:** J. M. Decundo, J. Mozo, D. S. Pérez Gaudio, G. Martínez, S. N. Dieguez, C. P. Bianchi, V. R. Eguía, M. L. Maté, J. P. Lirón, F. A. Amanto, A. L. Soraci

**Affiliations:** 1 Laboratorio de Toxicología, Depto. de Fisiopatología, Centro de Investigación Veterinaria de Tandil (CIVETAN), Facultad de Ciencias Veterinarias, Universidad Nacional del Centro de la Provincia de Buenos Aires, Tandil, Argentina; 2 Consejo Nacional de Investigaciones Científicas y Técnicas (CONICET), Ciudad Autónoma de Buenos Aires, Buenos Aires, Argentina; 3 Depto. de Producción Animal, Facultad de Ciencias Veterinarias, Universidad Nacional del Centro de la Provincia de Buenos Aires, Tandil, Argentina; 4 Comisión de Investigaciones Científicas de la Provincia de Buenos Aires (CIC-PBA), La Plata, Argentina; 5 Laboratorio de Endocrinología, Depto. de Fisiopatología, Centro de Investigación Veterinaria de Tandil (CIVETAN), Facultad de Ciencias Veterinarias, Universidad Nacional del Centro de la Provincia de Buenos Aires, Tandil, Argentina; 6 Laboratorio de Farmacología, Depto. de Fisiopatología, Centro de Investigación Veterinaria de Tandil (CIVETAN), Facultad de Ciencias Veterinarias, Universidad Nacional del Centro de la Provincia de Buenos Aires, Tandil, Argentina

**Keywords:** antibiotic alternatives, gut microbiota, intestinal integrity, phytogenic additives, prebiotics, weaned piglets

## Abstract

Early weaning is a critical stage in pig production, associated with intestinal and immunological stress that negatively impacts gut health and functionality. Given the global restrictions on antibiotic use, natural alternatives have gained increasing attention. This study aimed to evaluate the *in vivo* effects of short-chain fructooligosaccharides, essential oils and sodium humate on intestinal health in weaned piglets. Four dietary treatments were applied for 15 days to weaned piglets: a basal diet (control); short-chain fructooligosaccharides (scFOSs group); essential oils of *L. origanoide*s and *E. caryophyllata* with sodium humate (EOs-SH group); and a combination of scFOSs and EO of *Lippia origanoides* (scFOSs-EOLo group). Plasma cortisol and citrulline concentrations, gastrointestinal pH, intestinal histomorphology, intestinal disaccharidase activity, lymphocyte infiltration, goblet cells quantification, mucus bacterial adherence, volatile fatty acids, and microbiota composition were analyzed. No significant differences in cortisol concentrations were found among treatments (*p* = 0.514). EOs-SH group showed significantly higher citrulline levels (*p* < 0.01), indicating enhanced enterocyte function. The activity of lactase in the proximal jejunum (*p* = 0.03) and maltase in the duodenum (*p* = 0.045) increased in scFOSs and EOs-SH groups, suggesting improved digestive capacity. Mucus bacterial adherence was also greater in EOs-SH group (*p* = 0.039), reflecting enhanced mucosal protective quality. Gastrointestinal pH values were within physiological ranges for the age and intestinal segment evaluated. ScFOSs-EOLo showed a higher gastric pH than the control (*p* = 0.033), while no differences among treatments were observed in the remaining gastrointestinal regions (*p* > 0.05). No treatment effect was observed for villus height or villus height-to-crypt depth ratio, with shallower ileal crypts in EOs-SH and ScFOSs-EOLo groups (*p* = 0.003), indicating improved mucosal integrity. The lowest intraepithelial lymphocyte counts in the ileum were observed in scFOSs and EOs-SH groups (*p* = 0.033). Short-chain fatty acid concentrations did not differ statistically among treatments (*p* > 0.05). Microbiota analysis revealed that the EOs-SH treatment reduced potentially proinflammatory genera, including *Clostridium sensu stricto* 1, *Terrisporobacter*, *Prevotella* 115, and *Subdoligranulum*. scFOSs supplementation markedly increased *Lactobacillus*, consistent with its prebiotic effect (Padj<0.05 in all cases). In contrast, ScFOSs-EOLo treatment did not induce any relevant abundance microbial changes (Padj>0.05). All dietary treatments improved some intestinal health parameters, with more consistent responses observed in the treatment with EOs-SH. This supplementation enhanced intestinal integrity and functionality, offering a natural strategy to support gut health and resilience in antibiotic-free production systems.

## 1 Introduction

Modern swine production systems rely on early weaning to optimize productivity, but this practice consistently exposes piglets to high physiological and nutritional stress (1–3). Early weaning is universally recognized as a critical period in a pig’s life, marked by detrimental impacts on health and performance (4). The abrupt dietary transition from highly digestible, liquid sow milk to a more complex solid feed compromises gastrointestinal health. This stress, combined with the piglet’s immature digestive and immune systems, results in reduced feed intake (anorexia), compromised nutrient absorption, disorganized epithelial architecture, and an imbalanced gut microbiota homeostasis (dysbiosis) (4–6). These effects frequently lead to diarrhea, slow growth, and mortality, causing significant economic losses (5, 7).

Historically, pig farmers and veterinarians extensively used prophylactic antibiotics, including antibiotic growth promoters (AGPs), to control post-weaning infections and mitigate the detrimental effects of stress (8). However, the irrational and widespread application of these antimicrobials has triggered therapeutic failures due to the global threat of antimicrobial resistance and marked intestinal dysbiosis (9, 10). In response to rising public health concerns, stringent governmental regulations, such as the 2006 European Union ban on AGPs (11) and the 2019/4 regulation restricting the prophylactic use of antimicrobials in medicated feed (12), have mandated a search for effective alternatives.

This global restriction has spurred intense research into safe, sustainable, and natural nutritional strategies capable of supporting intestinal health and replacing antibiotics in swine diets. Promising alternatives include acidifiers, prebiotics, probiotics and phytobiotics (plant extracts and essential oils, EOs) (13–15).

Current strategies focus on incorporating specific compounds with established efficacy in enhancing gut health. Fructooligosaccharides (FOS) are non-digestible carbohydrates that resist hydrolysis by porcine intestinal enzymes. As established prebiotics, short-chain fructooligosaccharides (scFOSs) selectively stimulate the growth of beneficial bacteria, such as *Lactobacillus* spp. and *Bifidobacterium* spp. (16). Their fermentation by gut microbes yields beneficial short-chain fatty acids (VFAs), notably acetic and butyric acid, which are critical for colonocyte energy and intestinal barrier function maintenance. Dietary scFOSs consumption was shown to enhance intestinal integrity, reduce diarrhea index, and attenuate mucosal inflammation in weaned piglets (17). EOs derived from aromatic plants, such as oregano (*Lippia origanoides*; Lo) and clove (*Eugenia caryophyllata*; Ec), are rich in phenolic compounds like thymol, carvacrol, and eugenol. These compounds exert antibacterial and antioxidant effects, helping to dampen the negative metabolic impact of weaning stress on enterocytes (as evidenced by increased plasma citrulline concentrations). These formulations, particularly those combining EOs, have demonstrated improvements in intestinal architecture, as increased villus height (Vh) and ratio to crypt depth (Cd), digestive enzyme activity (13, 17) and microbiota modulation (17). Moreover, sodium humates (SH), components sometimes included in these formulations, possess high adsorptive capacity, potentially assisting in reducing diarrhea incidence and influencing gut ecology (17–19).

Research indicates that combining different alternatives to antibiotics, often encapsulated for targeted release, is a more robust strategy than using single additives to replace antimicrobial use entirely. Technologies such as microencapsulation and additive combinations have been shown to be more effective in enhancing immuno-antioxidant capacity, improving intestinal barrier function, and modulating hindgut microbiota structure (13, 20). Given the potential demonstrated by the functional ingredients previously mentioned, the objective of the present study was to evaluate the effects of three additives: scFOSs, a combination of EOs of Lo and Ec with SH, and a combination of scFOSs and EOLo, focusing on key indicators of intestinal health, morphology, metabolism, and microbiota modulation in weaned piglets.

## 2 Materials and methods

### 2.1 Animals, diets and experimental design

The study was conducted on a commercial pig farm with high sanitary status in Buenos Aires Province, Argentina. All animals were managed according to the farm’s routine procedures. Pigs handling and experimental procedures were approved by the Animal Welfare Committee of the University of the Center of Buenos Aires Province (Res. 087/02; FCV-UNCPBA; internal record 11/23) and by the Faculty of Veterinary Sciences of the University of Buenos Aires (internal record 2024/18) and were carried out in accordance with (21).

The trial consisted of two independent temporal replicates of 400 piglets each, totaling 800 clinically healthy, newly weaned animals (21 ± 2 days old, 50% castrated males and 50% females) with homogeneous body weight (5.76 ± 0.05 kg) and similar genetic background (Choice Genetics). Piglets were randomly allocated into four experimental groups (*n* = 100 per treatment per replicate) and housed in separate rooms within an environmentally controlled barn (22 ± 5 °C, light, dark cycle 12,12 h, relative humidity 45–65%), with *ad libitum* access to feed and water.

All groups received a commercial basal diet. During the first week post weaning, piglets consumed a nursery diet and during the second week post weaning a transition diet (Perfecto®, Biofarma S. A., Córdoba, Argentina). All nutrient requirements were supplied in compliance with the National Research Council (22). Both diets were based on extruded corn and wheat bran as the main cereal sources; soybean meal, soybean expeller, and extruded soybean as primary plant protein sources; and fish meal and plasma as animal protein sources. Soybean oil was used as the main fat source. Dairy by-products, including whole milk and whey powder, were included to support early post-weaning nutritional requirements. Diets were supplemented with a standard vitamin-mineral premix. Ingredient inclusion rates and detailed formulations remain proprietary to the manufacturer. Nutritional specifications, based on calculated formulation values provided by the producer, are presented in Table 1. Basal diets were supplemented as follows for 15 days:

**Table 1:** Composition of basal weaning diet.

Nutritional specifications	First-week feed	Second-week feed
Dry Matter (%)	92.50	92.50
Crude protein (%)	21.85	20.56
Fat (%)	5.80	6.53
Starch (%)	25.80	31.29
Crude Fiber (%)	1.50	1.65
Ash (%)	5.49	5.35
Calcium	0.74	0.90
Available phosphorus (%)	0.57	0.48
Metabolizable Energy (kcal)	3394.41 Kcal	3410.40 Kcal
Net Energy (kcal)	2436.35 Kcal	2489.17 Kcal
Lactose (%)	12.60	10.54
Digestible lysine (%)	1.52	1.39
Digestible methionine (%)	0.61	0.46
Methionine + cystine (%)	0.94	0.59
Digestible threonine (%)	0.98	0.84
Digestible tryptophan (%)	0.29	0.24
Digestible arginine (%)	1.28	1.27
Digestible valine (%)	0.98	0.92
Digestible isoleucin (%)	0.82	-
Digestible leucine (%)	1.61	-

Control group: Basal diet only;

scFOSs group: Basal diet supplemented with a formulation containing 40% scFOSs, included at 700 g/ton;

EOs-SH group: Basal diet supplemented with a microencapsulated formulation containing EOs (8% EOLo, 3% EOEc) and SH, included at 900 g/ton, where both EOs were pure extracts as obtained by distillation of the aromatic plants; and,

scFOSs-EOLo group: Basal diet supplemented with a formulation combining 34% of scFOSs and 16% of EOLo, included at 450 g/ton.

All dietary additives were kindly provided by Promitec SAS (Santander, Colombia) and included at their recommended doses, which fall within the range of inclusion levels previously reported and validated in the scientific literature (13, 17, 23).

Animal health status was monitored daily by trained personnel, who recorded the occurrence of diarrhea, behavioral alterations, and any observable abnormalities in feed or water intake. Growth performance parameters (body weight at days 40 and 70, average daily gain, feed conversion ratio) derived from this experimental system have been previously reported (23) and are therefore not included in the present manuscript.

### 2.2 Sample collection and processing

#### 2.2.1 Plasma

Blood samples were collected from 80 piglets individually identified with an ear tag (*n* = 20/treatment group), by venipuncture of the anterior vena cava on predefined post-weaning days (days 0, 4, 8, 12, and 15). Samples were collected in heparinized tubes, immediately centrifuged to obtain plasma, and stored at −20 °C until analysis. All blood sampling procedures started at 08:00 h and were completed within a maximum of 30 min to minimize handling-related variability.

#### 2.2.2 Gastrointestinal tract

At the end of the 15-day post-weaning trial, six ear tagged-piglets per group (three from each replicate) were randomly selected and euthanized by captive-bolt stunning followed by exsanguination via jugular severance. The gastrointestinal tract was immediately dissected, and tissue samples were collected without delay. A general assessment of gastrointestinal health was performed following the protocol described by Martínez et al. (15), with the specific analytical procedures detailed in the Analytical Methods section.

#### 2.2.3 Fecal samples

Fresh fecal samples were collected directly within the room by trained personnel wearing sterile gloves. Samples were obtained immediately after spontaneous defecation by gently collecting a small portion from the central area of the fecal mass using a sterile spoon, ensuring no contact with the pen floor or external surfaces. Individual samples were progressively pooled in sterile tubes to obtain a representative composite sample from 10 piglets housed in the same room. Pooled samples were immediately placed on ice and subsequently stored at −72 °C until DNA extraction. Sampling was performed at 0, 4, 8, 15, 22, and 39 days post-weaning for each treatment and replicate.

### 2.3 Analytical methods

#### 2.3.1 Plasma cortisol and citrulline concentrations

Plasma cortisol concentrations were used as an indicator of systemic stress (24, 25). Cortisol was quantified using a radioimmunoassay (RIA) kit (IM 1841, Beckman Coulter, Immunotech), following a protocol previously validated for porcine plasma (13, 26).

Plasma citrulline concentrations were assessed as a biomarker of intestinal integrity, enterocyte functional mass and metabolism (27–29). Citrullinemia was determined using High-Performance Liquid Chromatography with Fluorescence Detection (HPLC-FLD) after derivatization with o-phthalaldehyde (OPA). Separation was performed on a C18 column under gradient elution, with detection at 338 nm (excitation) and 425 nm (emission) (30). The analytical method exhibited excellent linearity (r^2^ > 0.999) within 0.5–20 μmol/L, an accuracy of 2.09%, and both repeatability and intermediate precision values <10% across all concentrations.

#### 2.3.2 Gastrointestinal pH

The pH was measured immediately upon dissection in the stomach (caudal portion), ileum, caecum, and colon using a pH meter (UP-25 Denver Instruments), calibrated in the range of pH 4 to 7 following manufacturerʼs instructions.

#### 2.3.3 Intestinal histomorphology, lymphocyte infiltration and goblet cells quantification

Segments (10 cm) from the mid-jejunum (1.5 m distal to the stomach) and ileum (20 cm proximal to the ileocecal valve) were collected, rinsed, fixed in 10% neutral buffered formalin or Bouin’s solution, embedded in paraffin, sectioned, and stained with Hematoxylin and Eosin (H/E) or Periodic Acid–Schiff (PAS). All histological slides were examined under a light microscope (Olympus BX40, Olympus Corporation, Tokyo, Japan) equipped with an image analysis system (ToupTek™ ToupView™, Anji, Zhejiang, China).

H/E-stained sections were used for morphometric evaluation. For each sample, the height and width of 50 villi and their corresponding crypts were measured. Vh, Cd, and the Vh: Cd ratio were calculated, and the Intestinal Absorptive Area (IAA) was estimated according to the mathematical model of Kisielinski et al. (31).

Intraepithelial lymphocytes (IELs) were quantified following the methodology of Hui et al. (32). Ten randomly selected fields per sample were analyzed at 40 × magnification, and counts were expressed as IELs per 100 enterocytes. Lymphocytic infiltration was classified as: 0 = normal (0–10 IELs/100 enterocytes), 1 = mild (10–15 IELs/100 enterocytes), 2 = moderate (15–20 IELs/100 enterocytes; indicative of subclinical chronic inflammation), and 3 = severe (>20 IELs/100 enterocytes; consistent with chronic inflammation and epithelial barrier impairment).

PAS-stained sections were used to quantify goblet cells in villi (vGC) and crypts (cGC), expressed as the number of goblet cells per 100 villi or 100 crypts. All histological measurements were performed by a single blinded evaluator.

#### 2.3.4 Intestinal disaccharidase activity

Segments from the duodenum, proximal jejunum (20 cm distal to the stomach), mid-jejunum, and ileum were collected. Each segment was opened along the mesenteric border, rinsed with saline solution, and the mucosa was carefully scraped off with a scalpel. A 1.00-g aliquot of mucosa was weighed, mixed with 2 mL of saline solution, and homogenized using an Ultra-Turrax followed by a Potter homogenizer. The homogenates were centrifuged at 4,825 × g for 10 min at 4 °C, and the obtained supernatant, considered the crude enzyme extract, was stored at −70 °C until analysis. Protein concentration was determined using the Bradford method with bovine serum albumin as the standard.

Sucrase, lactase, and maltase activities were quantified according to the Dahlqvist methodology (33), which measures glucose released after substrate hydrolysis. Briefly, homogenate supernatants were diluted and incubated for 1 h at 37 °C with an equal volume of 56 mmol/L sucrose, lactose, or maltose solutions prepared in sodium maleate buffer (pH 6.0). Glucose concentration in the reaction mixture was determined using a glucose oxidase–peroxidase assay (Sigma Chemical Co.) with O-dianisidine as the chromogen, and absorbance was read at 450 nm using a spectrophotometer (Shimadzu RF-5301PC). Disaccharidase activity was expressed as units per milligram of protein (U/mg protein), where one unit (U) represents the amount of enzyme that hydrolyzes 1 μmol of substrate per minute under the assay conditions.

#### 2.3.5 Mucus bacterial adherence

The adherence of pathogenic *Escherichia coli* O157: H7 to intestinal mucus was evaluated following the method described by Bai et al. (34). Ileal sections were opened along the mesenteric border, and mucus was carefully scraped off with a scalpel, leaving the underlying mucosa intact, and collected into sterile tubes. Samples (100 mg) were diluted in 1.5 mL of saline solution, homogenized, and centrifuged at 4,825 × g for 10 min at 4 °C. The resulting supernatant was filtered through 0.22-μm nylon membranes to obtain the crude mucus fraction containing glycoproteins responsible for bacterial adhesion.

Crude mucus was incubated with 10^3^ CFU/mL of *E. coli* O157: H7 for 30 min at 37 °C under continuous agitation. After centrifugation at 14,400 × g for 10 min at 4 °C, the pellet (containing adhered bacteria) was resuspended in 400 μL of saline solution and subjected to a low-speed centrifugation step (400 × g, 2 min, 4 °C) to separate adhered from non-adhered bacteria. Aliquots from both the pellet (adhered) and the supernatant (non-adhered) were plated on sorbitol MacConkey agar (Britania S. A.) and incubated aerobically for 24 h at 37 °C. Colony-forming units were counted, and results were expressed as the percentage of bacteria adhered to the intestinal mucus.

This approach provides a functional measure of intestinal mucus quality based on its bacterial binding capacity.

#### 2.3.6 Microbiota composition

Total bacterial DNA was isolated using the QIAamp® PowerFecal® Pro DNA Kit (Qiagen, Redwood City, CA, United States), and DNA concentration and purity were assessed with a Nanodrop spectrophotometer.

The V3-V4 hypervariable region of the bacterial 16S rRNA gene was amplified by PCR using primers 341F (CCTAYGGGRBGCASCAG) and 806R (GGACTACNNGGGTATCTAAT). Amplicons were purified, quantified with a Qubit™ fluorometer, and their integrity was verified using an Agilent 2,100 Bioanalyzer.

Barcoded libraries were sequenced on the Illumina NovaSeq 6,000 platform (Novogene, Durham, NC, United States). FASTQ files were processed in QIIME2, where reads were denoised and quality-filtered using the DADA2 plugin. Taxonomic assignment was performed using a naïve Bayes classifier trained on the SILVA v138 database, restricted to the V3-V4 region (35). Resulting feature tables, taxonomic annotations, phylogenetic trees, and metadata were imported into the Phyloseq R package (v1.42.0) (36) for downstream ecological analyses. Sequences identified as chloroplasts, mitochondria, or eukaryotes were discarded. Datasets were rarefied to 90% of the minimum sequencing depth prior to calculating alpha diversity and beta diversity metrics across samples. FASTQ files and associated metadata have been deposited in the CONICET institutional repository and are publicly available at https://datosdeinvestigacion.conicet.gov.ar/handle/11336/280356.

#### 2.3.7 Volatile fatty acids

VFAs were quantified following the method described by Jouany (37). Briefly, 1.00 g of cecal content was collected into sterile tubes containing phosphoric acid (4:1, wt/wt) for preservation and stored at −70 °C until analysis. VFAs were extracted with methanol, vortex-mixed, cold-centrifuged, and the supernatant was filtered through 0.22-μm membranes prior to chromatographic injection. Chromatographic analysis was performed using a Shimadzu GC-17A gas chromatograph (Kyoto, Japan) equipped with a flame ionization detector (FID) and a 30-m INNOWAX capillary column (Agilent, Santa Clara, CA, United States). Calibration curves were constructed using a mixture of certified VFAs standards (Supelco, Muskoka, ON, Canada) with 2-ethyl-butyric acid (Fluka, Charlotte, NC, United States) as the internal standard. The analytical method demonstrated optimum linearity (r^2^ > 0.995) over the range of 0.0625–9 mmol/L, with accuracy, repeatability, and intermediate precision values <10% for all analytes across concentrations.

### 2.4 Statistical analysis

All statistical analyses were conducted using R software (version 4.2.2) (38). Data were expressed as mean ± standard deviation (SD) as appropriate. Normality and homoscedasticity were assessed using the Shapiro–Wilk and Bartlett tests, respectively.

For variables derived from biological subsampling (blood parameters, intestinal histomorphology, enzyme activity, immune markers and VFAs), individual animals were considered the experimental units, while replicate was included as a blocking factor. For fecal microbiota analyses, the room-level pooled sample was considered the experimental unit.

Plasma cortisol and citrulline concentrations were analyzed using repeated-measures ANOVA, evaluating the effects of treatment, sampling day, and their interaction; when significant effects (*p* < 0.05) were detected, pairwise comparisons were performed using Tukey or Dunn tests, as applicable.

Variables evaluated at a single time point, including IAA, Vh: Cd, IELs, vGC and cGC, disaccharidase activities, mucus bacterial adherence, and cecal VFA concentrations, were analyzed using one way ANOVA, or Kruskal-Wallis test according to data distribution. Intestinal segments were analyzed individually.

For microbiota analysis, alpha diversity was assessed using the Shannon index, and group differences were evaluated with the Kolmogorov–Smirnov test. Beta diversity was assessed using Bray-Curtis and unweighted UniFrac distance matrices and visualized through Principal Coordinate Analysis (PCoA). Differential relative abundance at the phylum, class, order, family, and genus levels was performed using DESeq2 (39), applying a generalized linear model (GLM) that included sampling day as a blocking factor. When the GLM indicated significant effects, pairwise comparisons between treatments were conducted using Wald tests. Taxa were considered significantly different when showing base mean > 10% reads, |log_2_ fold change| (Log2FC) > 1, and adjusted *p* values (Padj) < 0.05. Taxa representing >1% relative abundance were prioritized for interpretation.

## 3 Results

Animals remained healthy, with consistent food and water consumption and no evidence of diarrhea throughout the trial.

### 3.1 Plasma biomarkers of stress and intestinal integrity

#### 3.1.1 Cortisol

No significant effect of treatment was detected (*p* = 0.514), and the sampling day × treatment interaction did not reach statistical significance (*p* = 0.065). In contrast, a clear effect of sampling day was observed (*p* < 0.01).

Across all groups, cortisol levels showed a transient decline at day 8 post-weaning, which exhibited the lowest mean concentration and differed significantly from all other sampling days. The highest cortisol concentrations were observed at weaning, with significant differences compared with days 8 and 12 post-weaning (Table 2).

**Table 2:** Cortisol and citrulline plasma concentrations at different sampling days for each treatment group.

Variables	Days	Group				Day mean	*p*-value		
		Control	scFOSs	EOs-SH	scFOSs-EOLo		Treatment	Day	Treatment x day
Cortisol (ng/mL)	0	73.49 ± 16.84	65.65 ± 18.74	63.61 ± 17.45	53.42 ± 15.17	64.60 ± 17.93^a^	0.514	<0.01	0.065
	4	57.13 ± 21.64	56.62 ± 15.32	54.62 ± 22.94	58.51 ± 19.87	56.23 ± 19.50^ac^			
	8	39.60 ± 18.29	30.81 ± 13.37	39.31 ± 23.47	52.55 ± 23.48	40.63 ± 21.10^b^			
	12	46.01 ± 11.56	51.42 ± 11.01	45.21 ± 17.09	52.09 ± 13.68	48.76 ± 13.47^c^			
	Group mean	53.06 ± 20.94	49.61 ± 19.23	50.18 ± 21.98	54.14 ± 18.43				
Citrulline (μM)	0	84.47 ± 19.11	78.66 ± 14.89	91.80 ± 12.26	85.07 ± 13.47	85.01 ± 16.20^a^	<0.01	<0.01	0.296
	4	48.89 ± 11.88	54.37 ± 9.427	61.40 ± 10.43	56.45 ± 12.72	55.26 ± 11.85^b^			
	8	56.38 ± 9.22	56.58 ± 11.11	71.43 ± 16.68	57.37 ± 8.468	60.48 ± 9.85^b^			
	12	66.21 ± 10.92	64.54 ± 11.53	85.65 ± 19.37	67.95 ± 20.13	71.21 ± 10.85^c^			
	15	74.38 ± 14.20	74.74 ± 18.82	94.71 ± 14.83	77.51 ± 14.73	80.37 ± 14.85^a^			
	Group mean	65.88 ± 18.22^a^	65.79 ± 16.48^a^	80.99 ± 19.88^b^	68.87 ± 18.02^a^				

Different superscript letters in the mean row or mean column indicate statistically significant differences (*p* < 0.05). Control, basal diet group; scFOSs, short-chain fructooligosaccharides group; EOs-SH, essential oils of *L. origanoides* and *E. caryophyllata* with sodium humate group; scFOSs-EOLo, combination of scFOSs and EOs of *L. origanoides* group.

#### 3.1.2 Citrulline

Plasma citrulline concentrations showed significant effects of treatment and sampling day (both p < 0.01), whereas their interaction was not significant (*p* = 0.246). Piglets in EOs-SH group exhibited higher citrullinemia than all other groups throughout the study period (Table 2).

**Figure 1:**
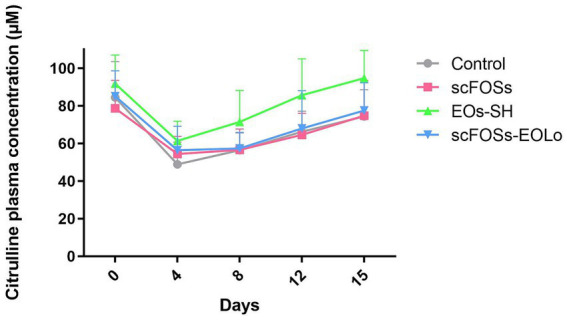
Citrulline plasmatic profile for each treatment group.

Across days, citrulline concentrations decreased markedly on days 4 and 8, which differed significantly from the remaining sampling points. By day 12, citrulline values began to recover and were statistically distinct from those observed on days 0, 4, 8, and 15. On day 15, citrulline concentrations returned to levels comparable to baseline (day 0; Figure 1).

### 3.2 Gastrointestinal pH along the digestive tract

All pH values fell within the expected physiological range for pigs of this age and for the corresponding gastrointestinal segments. In the stomach, scFOSs-EOLo group exhibited a significantly higher pH compared with the control group. No differences between treatments were detected in the ileum, caecum, or colon (Table 3).

**Table 3:** Gastrointestinal pH values (mean ± SD) in each segment by treatment group.

Parameter	Segment	Group				*p*-value
		Control	scFOSs	EOs-SH	scFOSs-EOLo	
pH	Stomach	2.82 ± 0.89^a^	3.46 ± 0.63^ab^	2.94 ± 0.73^ab^	4.23 ± 1.02^b^	0.033
	Ileum	7.08 ± 0.27	7.17 ± 0.11	7.00 ± 0.24	7.16 ± 0.16	0.496
	Cecum	6.16 ± 0.20	5.97 ± 0.30	5.99 ± 0.34	5.89 ± 0.24	0.449
	Colon	6.84 ± 0.47	5.95 ± 0.37	6.56 ± 0.69	6.65 ± 0.59	0.063

Different superscript letters within a row indicate statistically significant differences (*p* < 0.05). Control, basal diet group; scFOSs, short-chain fructooligosaccharides group; EOs-SH, essential oils of *L. origanoides* and *E. caryophyllata* with sodium humate group; scFOSs-EOLo, combination of scFOSs and EOs of *L. origanoides* group.

### 3.3 Intestinal histomorphology

No treatment effects were detected for Vh, IAA, or the Vh: Cd ratio in either the jejunum or the ileum. A treatment effect was observed only for Cd in the ileum, where the EOs-SH and scFOSs-EOLo groups exhibited significantly lower values compared with the control group (Table 4).

**Table 4:** Histomorphological, mucosal immune and barrier-related parameters (mean ± SD) in the Jejunum and Ileum for each treatment group.

Segment	Parameter		Group				*p*-values
			Control	scFOSs	EOs-SH	scFOSs-EOLo	
Jejunum	Histomorphological	*Vh*	344.94 ± 64.36	350.55 ± 77.03	330.76 ± 57.80	353.29 ± 35.51	0.919
		*Cd*	108.57 ± 15.90	106.87 ± 11.29	89.82 ± 8.25	96.27 ± 19.15	0.105
		*AAI*	6.73 ± 0.90	6.63 ± 0.85	6.47 ± 0.58	6.94 ± 0.45	0.724
		*Vh; Cd*	3.21 ± 0.62	3.37 ± 1.11	3.69 ± 0.61	3.80 ± 0.87	0.559
	Mucosal immune and barrier-related	*IELs*	2.58 ± 1.22	2.12 ± 1.58	1.87 ± 0.83	2.27 ± 1.02	0.541
		*vGC*	1404.48 ± 363.33	1371.72 ± 98.60	1156.97 ± 312.45	1062.11 ± 245.69	0.119
		*cGC*	1409.33 ± 487.63	1479.04 ± 460.97	1457.64 ± 607.61	1277.98 ± 313.38	0.910
Ileum	Histomorphological	*Vh*	272.59 ± 30.56	259.52 ± 40.98	292.86 ± 84.75	278.58 ± 34.46	0.450
		*Cd*	108.57 ± 15.90^a^	97.15 ± 15.59^ab^	84.82 ± 23.97^b^	89.92 ± 10.72^b^	0.03
		*AAI*	5.50 ± 0.84	5.16 ± 0.67	5.98 ± 5.68	5.38 ± 0.69	0.855
		*Vh: Cd*	2.53 ± 0.44	2.76 ± 0.76	3.18 ± 0.38	3.16 ± 0.66	0.180
	Mucosal immune and barrier-related	IELs	3.30 ± 2.17^ab^	1.22 ± 0.50^c^	1.83 ± 0.96^bc^	3.43 ± 1.75ª	0.033
		vGC	1378.49 ± 278.93	1368.02 ± 494.24	1511.60 ± 424.09	1566.38 ± 481.94	0.813
		cGC	1875.33 ± 479.15	1909.33 ± 350.13	1966.50 ± 624.54	1924.17 ± 349.08	0.750

Different superscript letters within a row indicate statistically significant differences (*p* < 0.05). Control, basal diet group; scFOSs, short-chain fructooligosaccharides group; EOs-SH, essential oils of *L. origanoides* and *E. caryophyllata* with sodium humate group; scFOSs-EOLo, combination of scFOSs and EOs of *L. origanoides* group; Vh, villus height; Cd, crypt depth; IAA, intestinal absorptive area; Vh: Cd, villus height to crypt depth ratio; IELs, intraepithelial lymphocytes; vGC, goblet cells in villi; cGC, goblet cells in crypt.

### 3.4 Intestinal disaccharidase activity

**Figure 2:**
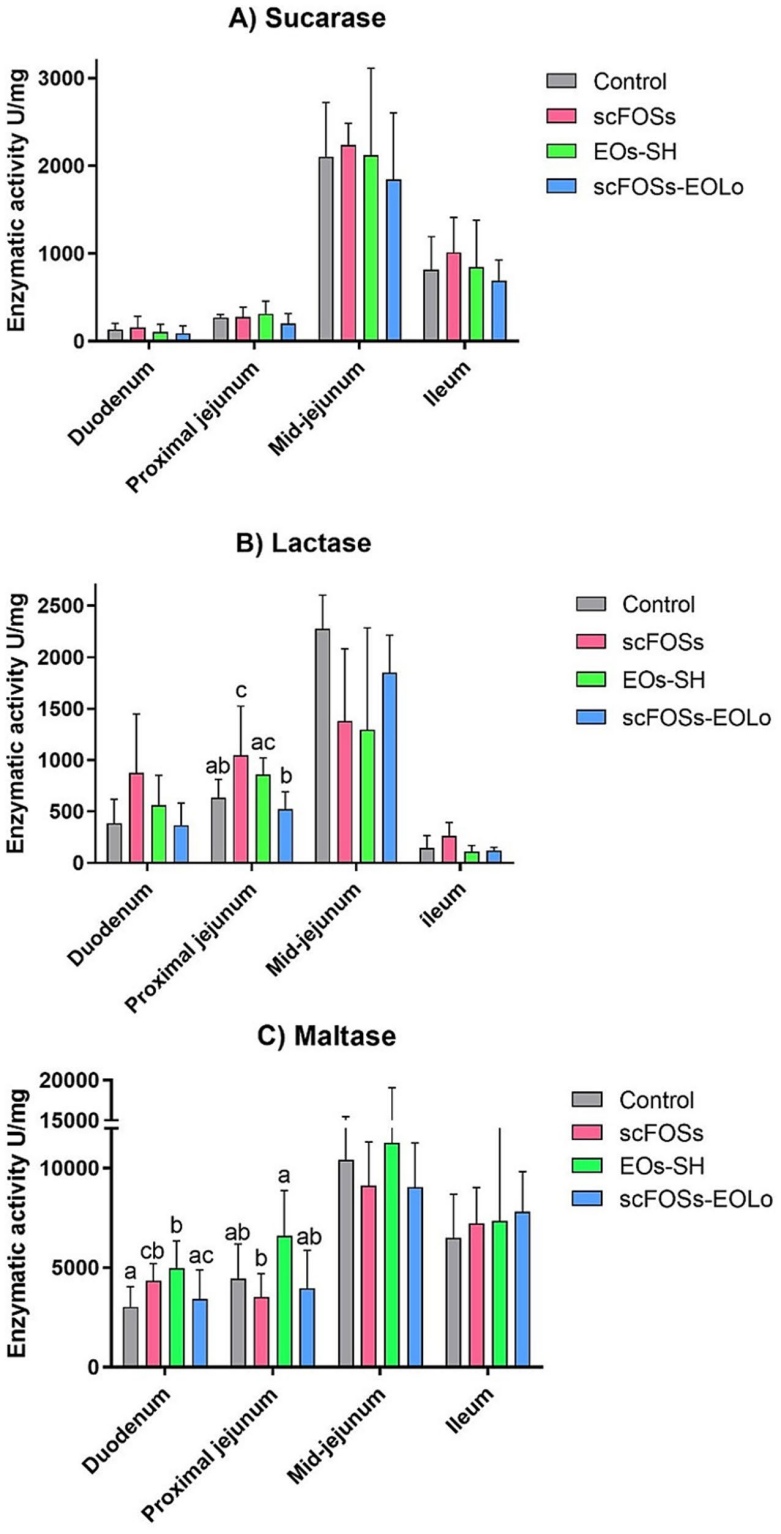
Mean (± SD) sucrase **(A)**, lactase **(B)**, and maltase **(C)** activities across intestinal segments (duodenum, proximal jejunum, mid-jejunum and ileum). Different letters (a–c) indicate statistically significant differences among treatment groups within each intestinal segment (*p* < 0.05).

No treatment effect was detected for sucrase in any of the intestinal segments evaluated (*p* > 0.05; Figure 2A).

For lactase, a treatment effect was observed only in the proximal jejunum (*p* = 0.030). In this segment, scFOSs group exhibited significantly higher enzymatic activity than control group and scFOSs-EOLo group, while EOs-SH group also showed higher activity than scFOSs-EOLo group (Figure 2B). In the remaining intestinal segment, no significant differences among treatments were detected (*p* > 0.05).

Regarding maltase, significant differences were detected in the duodenum (*p* = 0.045), where EOs-SH group presented greater activity than both the control group and scFOSs-EOLo group. scFOSs group also showed higher duodenal maltase activity than the control group. In addition, a treatment effect was observed for maltase in the proximal jejunum (*p* = 0.040), where EOs-SH group achieved the highest activity values, significantly greater than those of the scFOSs group (Figure 2C). No significant differences among treatments were observed in the remaining intestinal segment evaluated (*p* > 0.05).

### 3.5 Mucosal immune and barrier-related parameters

#### 3.5.1 IEL

Across all groups and intestinal regions, IEL counts fell within the normal range (0–10 IELs per 100 enterocytes). In the mid-jejunum, no differences among treatments were detected. In the ileum, the lowest IEL counts were observed in the scFOSs group, which differed significantly from both the scFOSs-EOLo and control groups. The EOs-SH group showed similarly low IEL values to those observed in the scFOSs group and differed significantly from the scFOSs-EOLo group (Table 4).

#### 3.5.2 vGC and cGC

No treatment effect was observed on vGC or cGC counts in either the jejunum or ileum (Table 4).

#### 3.5.3 Mucus bacterial adherence

**Figure 3:**
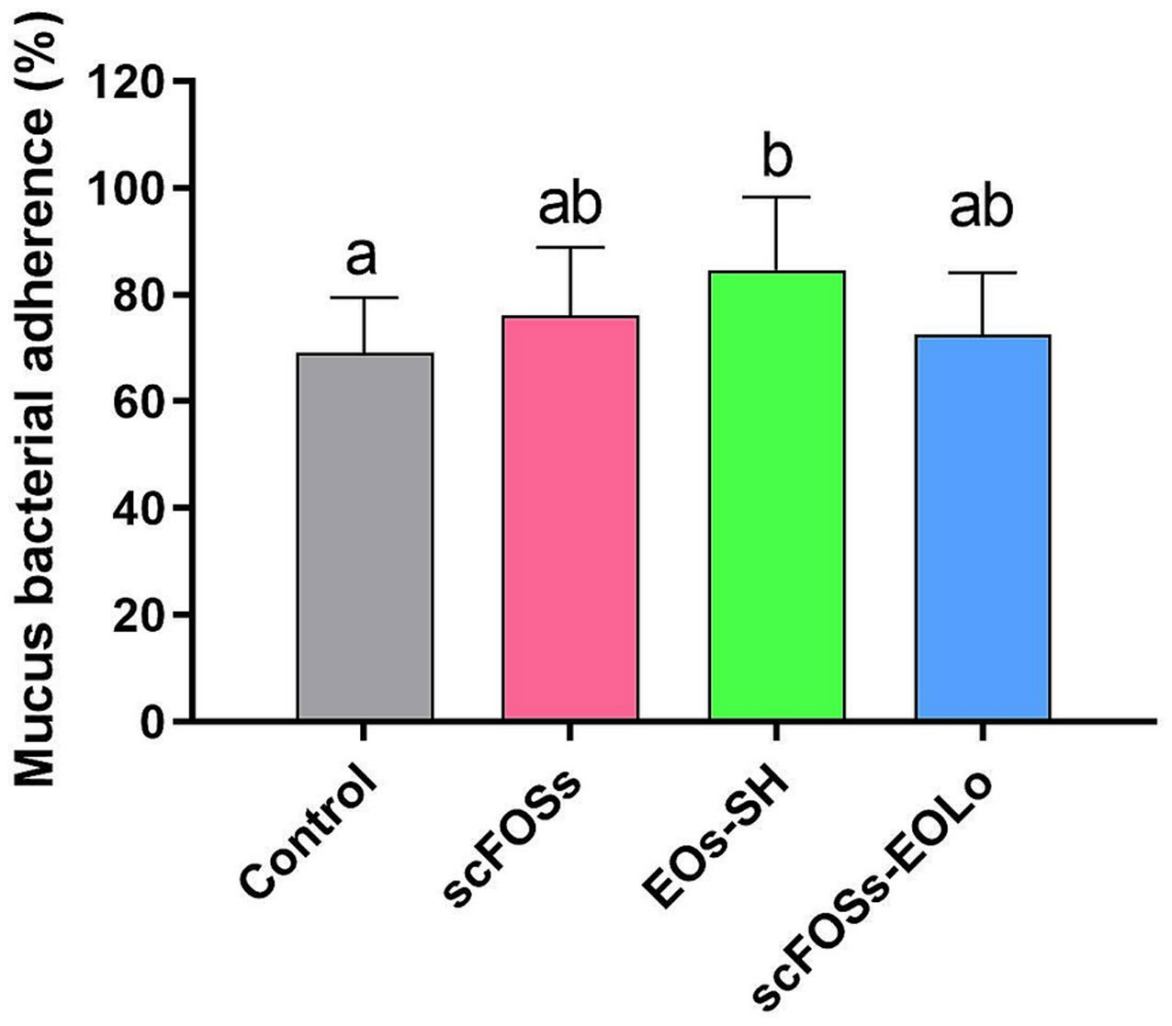
Mean percentage (± SD) of *E. coli* O157: H7 adherence to intestinal mucus. Different letters (a–c) indicate statistically significant differences among groups (*p* < 0.05).

A significant treatment effect was detected for bacterial adherence to intestinal mucus (*p* = 0.039). Piglets in EOs-SH group exhibited a significantly higher percentage of *E. coli* O157: H7 adhesion compared with the control group (Figure 3).

### 3.6 Microbiota composition and diversity

**Figure 4:**
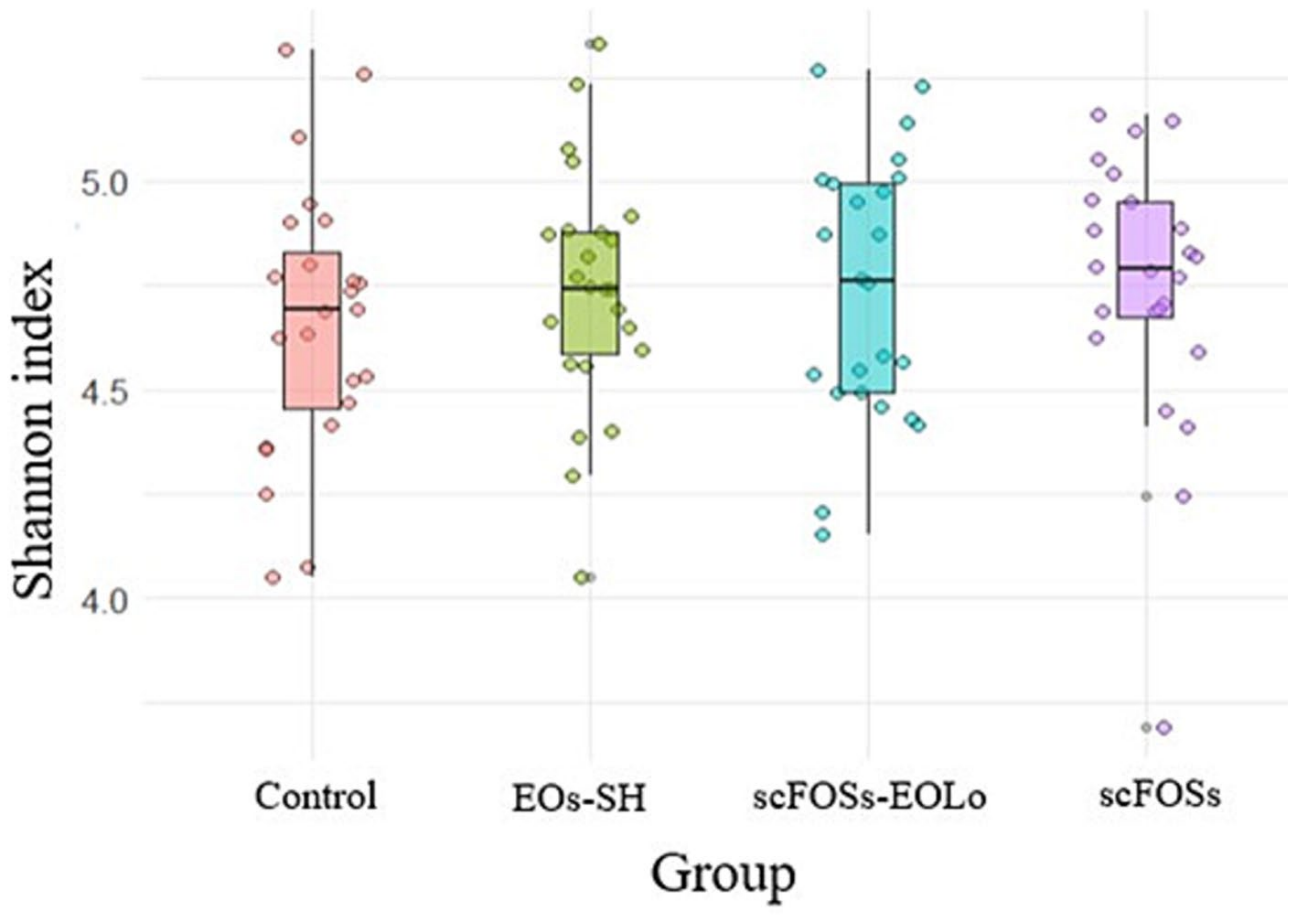
Alpha diversity (Shannon index) of the fecal microbiota across dietary treatments.

The microbiome quality assessment indicated that library sizes ranged from 76,942 to 247,076 clean reads per sample. All samples met the required quality standards for downstream bioinformatic analyses (Supplementary Table S1). Alpha diversity showed similar Shannon index values for all piglet groups, ranging from 4.69 ± 0.33 to 4.79 ± 0.01 (*p* > 0.05; Figure 4).

**Figure 5:**
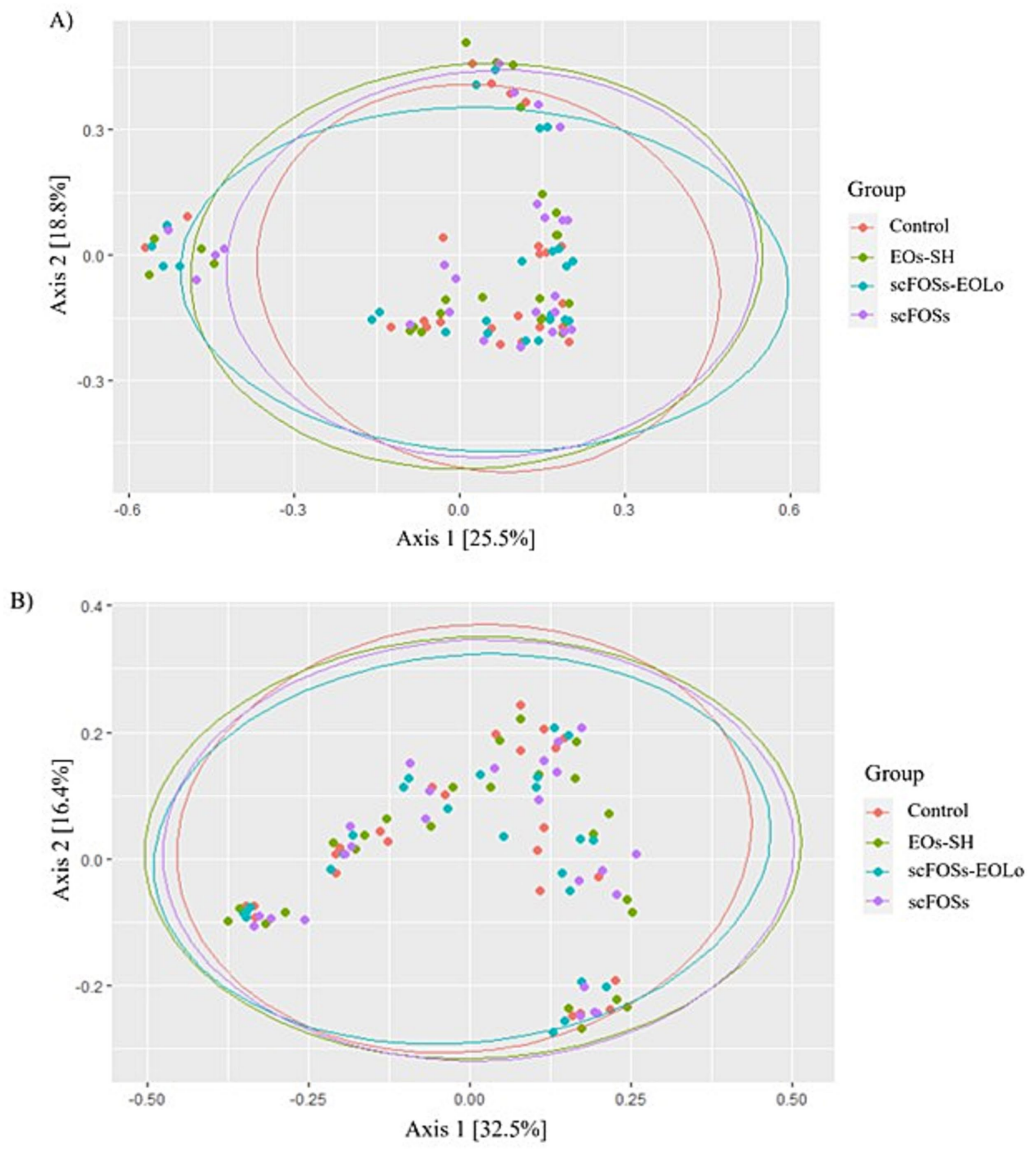
Principal coordinates analysis (PCoA) plots based on **(A)** Bray-Curtis and **(B)** unweighted UniFrac distance metrics for evaluated groups.

The PCoA plots for beta diversity analysis showed overlapping clusters (Figure 5), suggesting that the microbiome structure was similar across the piglet groups.

Despite the overall similarity in community structure, microbiota profiling revealed treatment-dependent shifts in bacterial relative abundance compared with the control group. Supplementation with the EOs-SH significantly decreased several members of the order *Clostridiales* (log2FC: −1.00; Padj 0.043), as well as the families *Peptostreptococcaceae* (log2FC: −1.11; Padj: 0.032), and *Clostridiaceae* (log2FC: −1.06; Padj: 0.045), while increasing the order *Methanobacteriales* (log2FC: 1.79; Padj: 0.043). At the genus level, EOs-SH significantly reduced taxa associated with proinflammatory activity, including *Clostridium sensu stricto 1* (log2FC: −2.22; Padj: 0.010), *Terrisporobacter 6* (log2FC: −1.59; Padj: 0.020), *Prevotella 115* (log2FC: −1.91; Padj: 0.021), and *Subdoligranulum* (log2FC: −1.74; Padj: 0.040).

In contrast, scFOSs supplementation induced a marked enrichment of the genus *Lactobacillus 27* (log2FC: 9.23; Padj<0.001), along with increases in *Streptococcus 12* (log2FC: 3.25; Padj: 0.002), *Methanosphaera 2* (log2FC: 4.40; Padj: 0.006). No significant changes in microbial relative abundance were detected in the combination treatment (scFOSs-EOLo), in which all taxa remained comparable to the control group (Padj > 0.05).

### 3.7 VFAs

No statistically significant differences were detected among treatment groups for any of the individual VFAs analyzed (acetic, propionic, butyric, total). Although not statistically different, EOs-SH group showed numerically higher concentrations of each VFA and of total VFAs compared with the other groups (Table 5).

**Table 5:** Mean (± SD) concentrations of volatile short-chain fatty acids (VFAs) in cecal content.

Parameter		Group				*p*-values
		Control	scFOSs	EOs-SH	scFOSs-EOLo	
VFA (mmol/L)	Acetic	80.40 ± 13.05	76.68 ± 9.80	89. 07 ± 7.90	79.29 ± 12.71	0.268
	Propionic	19.07 ± 5.16	20.64 ± 7.96	23.35 ± 2.41	20.92 ± 6.43	0.277
	Butyric	8.89 ± 3.31	7.85 ± 1.62	11.34 ± 4.22	10.20 ± 3.30	0.296
	Total	110.25 ± 20.58	105.81 ± 11.06	125.80 ± 9.76	110.59 ± 16.00	0.141

Control, basal diet group; scFOSs, short-chain fructooligosaccharides group; EOs-SH, essential oils of *L. origanoides* and *E. caryophyllata* with sodium humate group; scFOSs-EOLo, combination of scFOSs and EOs of *L. origanoides* group; VFA, volatile fatty acids.

## 4 Discussion

Early weaning imposes a multifactorial stress response in piglets with pronounced alterations in gastrointestinal morphology and immune function (1, 2). Since the global intensification of swine production systems made early weaning a standard practice, numerous studies have focused on optimizing post-weaning diets to mitigate these detrimental effects. Without appropriate nutritional and management strategies, the acute post-weaning phase consistently results in impaired performance and significant economic losses (4, 40).

In this context, the search for natural feed additives capable of sustaining intestinal integrity, enhancing digestive functionality, and reducing reliance on antibiotics has intensified. Among these, scFOSs have been widely evaluated as prebiotic ingredients capable of promoting beneficial bacterial populations, improving enterocyte maturation, and strengthening mucosal barrier function. Their fermentation by commensal microbiota resulted in production of VFA, modulation of intestinal pH, and competitive exclusion of pathogens, collectively contributing to improved gut health in weaned piglets (41, 42). Plant-derived EOs, particularly those rich in phenolic compounds such as thymol, carvacrol, and eugenol, including extracts from Lo and Ec, have gained substantial interest as natural antimicrobial and anti-inflammatory tools for swine nutrition.

When included as dietary additives, EOs were shown to improve gastrointestinal function and mitigate the adverse consequences of weaning stress (10, 43, 44). Their bioactive constituents modulated intestinal redox homeostasis, suppressed opportunistic pathogens, and favorably influenced the microbial community structure in weaned pigs (13). Nonetheless, EOs vary widely in biological activity depending on botanical source, extraction method, degree of microencapsulation, and administration strategy (45). Consequently, each formulation must be specifically assessed under real production conditions to determine its efficacy and applicability. Humic substances, including SH, have emerged as promising functional additives in swine nutrition. Their physicochemical properties enable toxin binding, modulation of oxidative stress, and immunomodulatory activity, thereby enhancing gastrointestinal epithelial resilience (46, 47). In young pigs, SH supported mucosal recovery during the post-weaning transition by increasing antioxidant defenses and attenuating inflammatory signaling, notably through reductions in pro-inflammatory cytokines such as TNF-*α*, IL-6, and IL-1β (18, 19). Concomitantly, SH contributed to improved intestinal barrier integrity via the upregulation of tight junction proteins, including occludin and claudins. These effects were accompanied by a favorable modulation of gut microbiota, characterized by increased abundance of beneficial genera and reduced proliferation of enteropathogenic bacteria, collectively reinforcing intestinal homeostasis (19).

Together, these additive classes represent distinct but potentially complementary strategies to mitigate the digestive and immune challenges experienced by piglets during weaning. The present study evaluated the effects of three functional dietary strategies, compared with a basal diet (control group): scFOSs, a blend of EOs with SH (EOs-SH), and a combination of scFOSs and EOLo, on physiological, biochemical, microbiological, and mucosal parameters in piglets during the early post-weaning phase.

The post-weaning phase is characterized by pronounced neuroendocrine activation, with cortisol serving as a key biomarker of the systemic response to environmental, nutritional, and social challenges (25, 29). In the present study, all piglet groups exhibited similar plasma cortisol concentrations throughout the sampling period, indicating that none of the supplementation strategies noticeably modulated the systemic stress response. Notably, when sampling days were considered, the highest cortisol values were observed at weaning (Table 2), highlighting the abrupt transition from sow-dependent to independent feeding as the period of greatest physiological stress. Thereafter, cortisol concentrations showed a clear decline by day 8 post-weaning, which likely reflects the natural attenuation of hypothalamic–pituitary–adrenal axis activation during the adaptation period (48).

Plasma citrulline dynamics clearly reflected the transient impairment and subsequent recovery of enterocyte functional mass typically associated with the post-weaning period. Across days, citrulline concentrations declined markedly on days 4 and 8, mirroring the acute suppression of enterocyte function widely documented during early weaning stress (13, 27). From day 12 onward, citrulline levels progressively increased and returned to baseline by day 15, indicating recovery of mucosal functionality. These findings support the use of citrulline as a sensitive indicator of mucosal integrity and enterocyte mass in swine (29).

The enhanced citrulline concentrations observed in EOs-SH-supplemented piglets may be biologically explained by known properties of the EO components. Molecules such as carvacrol and thymol (present in Lo) and eugenol (derived from Ec) have been shown to modulate key transcriptional pathways involved in oxidative and inflammatory responses, including NF-κB and Nrf2 signaling (49, 50). Through the suppression of oxidative stress, scavenging of reactive oxygen species, and attenuation of inflammation and apoptosis (10, 49), these compounds help preserve enterocyte viability and metabolic performance during the critical early post-weaning window. Collectively, the consistent elevation of citrulline in EOs-SH group suggests that this formulation was particularly effective at mitigating the early decline in enterocyte function and supporting intestinal mucosal resilience.

Gastrointestinal pH values remained within the expected physiological range for piglets of this age and intestinal segment, in agreement with previous reports in weaned pigs (13, 51). No treatment effects were observed in the ileum, caecum, or colon, indicating that the evaluated supplements did not induce substantial shifts in luminal acidity in the distal gut. The only significant difference was detected in the stomach, where piglets receiving the combined scFOSs-EOLo supplementation exhibited a moderately higher gastric pH compared with the control group. Functionally, a slight reduction in gastric acidity may help preserve mucosal integrity and enhance the survival of beneficial bacteria transiting through the stomach. However, excessive increases in gastric pH could theoretically impair the activation of pepsin and early protein digestion, as pepsin requires an acidic environment to function optimally (52). In the present study, the magnitude of the change was modest and unlikely to compromise digestive physiology. Overall, the data indicate that none of the supplementation strategies produced disruptive shifts in gastrointestinal pH, supporting their safety and compatibility with normal digestive function in early-weaned piglets.

Digestive enzyme activity displayed clear segment-specific patterns, indicating that the supplements differentially influence brush-border function along the small intestine. Compared with the control group, scFOSs supplementation increased lactase activity in the proximal jejunum, whereas both scFOSs and the EOs-SH formulation enhanced duodenal maltase activity. These responses suggest improved brush-border maturation or modulation of enterocyte turnover, consistent with the well-described trophic effects of prebiotics and phytogenic compounds on the intestinal epithelium (10, 53–55).

The increased maltase activity observed in the EOs-SH group aligned with the higher plasma citrulline concentrations recorded in these animals, supporting the interpretation that this supplement favored greater preservation of enterocyte functional mass during the acute post-weaning phase. A similar enhanced brush-border enzymatic activity with Lo and Ec EOs has been previously reported by Diéguez et al. (13). In contrast, the combination of scFOSs-EOLo did not enhance digestive enzyme activity and consistently showed lower values than those obtained with individual supplements. This lack of additivity suggests potential interference at the metabolic or microbial level, such as competition between fermentable substrates and antimicrobial phytochemicals, that may limit epithelial adaptation rather than promote synergistic effects. Taken together, the enzymatic profile indicates that scFOSs and EOs-SH exert beneficial but non-synergistic effects on mucosal maturation, and that combining both supplements may attenuate rather than potentiate their functional benefits.

Histomorphometric parameters exhibited limited variation in dietary treatments in this study. No significant effects were detected for IAA or for the Vh: Cd ratio, and most values remained within the expected physiological range for piglets of this age. A reduction in ileal Cd was observed in EOs-SH and scFOSs-EOLo groups compared with the control group, which may reflect decreased proliferative turnover or attenuated inflammatory remodeling of the mucosa.

Phytogenic feed additives, particularly formulations containing thymol, carvacrol, and eugenol, can promote beneficial architectural changes such as reduced Cd, increased Vh, and improved Vh: Cd ratios (53, 54). Similar reductions in Cd and improvements in villus morphology have been reported by Diéguez et al. (13) in piglets supplemented with Lo and Ec EOs. However, unlike those studies, our results did not reveal a consistent treatment-driven improvement across all histomorphological indices. The decrease in Cd in EOs-SH and scFOSs-EOLo animals may suggest a mild trophic or anti-inflammatory effect of these additives, but the overall mucosal architecture remained largely unchanged. This limited response could be related to the duration of supplementation or the intensity of the post-weaning challenge.

Across all groups, IEL counts remained within the physiological range for healthy piglets (0–10 IELs/100 enterocytes), yet meaningful differences emerged within this normal window. The lowest IEL densities in the ileum were observed in piglets supplemented with scFOSs, while the EOs-SH group exhibited comparably low values, although these did not differ significantly from the control group. Lower IEL counts are widely interpreted as indicative of reduced basal immune activation, improved epithelial stability, and diminished inflammatory signaling (56, 57). These findings align with the known anti-inflammatory and antioxidative properties of scFOSs (58) and phytogenic compounds such as thymol, carvacrol, and eugenol, which attenuated NF-κB-mediated responses and mitigated epithelial stress (10, 55). The scFOSs-EOLo formulation did not reproduce these benefits, suggesting that the immunomodulatory effects observed with scFOSs or EOs-SH were not additive and that co-administration of distinct bioactive compounds does not necessarily enhance mucosal immune regulation.

A significant treatment effect was detected for bacterial adherence to intestinal mucus, with the EOs-SH group exhibiting a higher percentage of *E. coli* O157: H7 adhesion compared with the control. Although *in vitro* adhesion assays do not necessarily predict pathogen colonization *in vivo*, they are sensitive indicators of biochemical modifications in the mucus layer, such as changes in glycan composition, mucin sulphation, or cross-linking. Similar increases in pathogen adhesion have been described in piglets supplemented with EOs, where phytogenic compounds stimulated mucin biosynthesis and altered mucus chemistry (59–62). Thus, the increased adhesion observed in EOs-SH piglets may reflect enhanced mucus complexity or secretion rather than a compromised barrier.

Goblet cell density is commonly considered an indicator of mucus production and has been associated with mucus barrier function and bacterial adhesion (63, 64). However, in the present study, no treatment-related differences were observed in goblet cell numbers, indicating that the increased bacterial adhesion to mucus in EOs-SH piglets was not associated with changes in goblet cell abundance. These findings reinforce the notion that mucus quality and functionality are not solely determined by goblet cell density, but are strongly influenced by qualitative, biochemical, and structural properties of the mucus layer, including mucin composition, organization, and epithelial secretory responses, as previously mentioned (65, 66). Indeed, diet-induced alterations in mucin composition, glycosylation, viscosity, or secretion dynamics may occur independently of goblet cell numbers and can modulate bacterial-mucus interactions and intestinal permeability (67–70).

When viewed alongside the other mucosal parameters, a coherent pattern emerges. Piglets supplemented with scFOSs exhibited the lowest ileal IEL counts, indicating reduced baseline epithelial immune activation. Similar values were achieved with EOs-SH supplementation, although no statistically significant differences from the control group were observed. Overall, the combined findings suggest that the additive-induced changes in mucus properties, particularly in the EOs-SH group, occurred in the context of low immune activation and preserved mucosal integrity. In this regard, previous studies highlight that improvements in mucosal barrier function induced by phytogenic compounds are often associated with broader regulatory effects on the intestinal ecosystem, including modulation of host–microbe interactions (17).

Microbiota diversity and composition were interpreted in relation to the intestinal and immunological outcomes observed in the present study. At the community level, the absence of significant differences in alpha diversity and the overlapping patterns observed in beta diversity analyses indicate that the dietary interventions did not induce broad restructuring of the microbial ecosystem. Instead, the treatments promoted selective shifts in specific bacterial taxa with functional relevance. Such targeted modulation is particularly expected during the early post-weaning period, which is characterized by high microbiota instability and marked inter-individual variability.

In line with this conceptual framework, microbiome analysis revealed clear, treatment-specific shifts that aligned with the functional and immunological patterns observed in the present study. Supplementation with EOs-SH produced the most pronounced ecological modulation, significantly reducing several genera commonly associated with proinflammatory or dysbiotic states, including *Clostridium sensu stricto 1*, *Terrisporobacter*, *Prevotella 115*, and *Subdoligranulum*. These taxa have been described as markers of intestinal dysbiosis or mucosal stress in pigs, often enriched under fermentative imbalance, epithelial perturbation, or immune activation. Their reduction therefore suggests a microbiota configuration less conducive to inflammatory signaling. These findings are consistent with previous reports describing the modulatory effects of phytogenic compounds on gut microbial communities. Similar shifts in microbial profiles, characterized by a reduction of *Firmicutes* groups linked to fermentative by-products, epithelial stress, and mucosal inflammation, were reported by Ángel-Isaza et al. (17), together with improvements in gut barrier function and immune indices. In line with this framework, the microbial changes observed in the EOs-SH group coincided with lower ileal IEL counts and preserved mucosal architecture, supporting a coordinated reduction in epithelial immune activation.

In contrast, scFOSs supplementation induced a robust enrichment of *Lactobacillus*, a hallmark response to prebiotic oligosaccharides. Expansion of *Lactobacillus* populations has been consistently associated with enhanced epithelial integrity, improved mucin metabolism, increased immune tolerance, and the production of beneficial metabolites in weaned piglets (16, 71, 72). Accordingly, the increased abundance of *Lactobacillus* observed in scFOSs-fed piglets in the present study was accompanied by reduced IEL density, supporting an improved mucosal immune equilibrium during the post-weaning period.

In addition to *Lactobacillus* enrichment, scFOSs supplementation was associated with increased relative abundance of *Streptococcus* and *Methanosphaera*. These taxa are frequently linked to enhanced carbohydrate fermentation, cross-feeding interactions, and hydrogen utilization within the intestinal ecosystem (71, 73). Although their functional roles in early-weaned piglets remain less clearly defined, such microbial shifts are consistent with a transition toward a more active saccharolytic fermentative environment rather than a pro-inflammatory microbial profile, as previously described in prebiotic-supplemented piglets (16, 74). Taken together, these treatment-specific microbial shifts-characterized by a reduction of taxa associated with proinflammatory or dysbiotic states in the EOs-SH group and the enrichment of *Lactobacillus* in scFOSs-supplemented piglets-provide a biologically coherent link with the observed improvements in mucosal immune tone, digestive enzyme activity, and markers of enterocyte functionality. These findings reinforce the concept that targeted microbial modulation, rather than large-scale diversity shifts, can meaningfully support intestinal maturation during the early post-weaning transition.

Notably, the combined supplementation of scFOSs and EOs-Lo did not result in significant microbial modulation, despite the clear effects observed when each additive was administered individually. This lack of response parallels the attenuated effects detected for digestive enzymes, mucus-related parameters, and mucosal immune markers in the combination group. Non-additive or antagonistic interactions between prebiotic-driven saccharolytic niches and the antimicrobial or regulatory effects of phytogenic compounds may underlie this outcome. Similar constraints have been described when functional feed components target overlapping metabolic pathways or compete for ecological niches (17).

In the present study, no statistically significant differences were detected among treatments for any individual or total VFAs, although the EOs-SH group consistently showed numerically higher concentrations compared with the other groups. These findings suggest that, under the conditions evaluated, dietary modulation of microbial fermentation was limited. Importantly, the absence of statistical significance is consistent with previous short-duration post-weaning studies. Consistent with the present findings, no differences in cecal VFAs concentrations between supplemented and control piglets were observed by Decundo et al. (51) under a 15-day experimental period similar to that applied in this study, suggesting that early post-weaning dynamics and high inter-individual variability may mask treatment effects during this acute window. By contrast, longer supplementation periods have demonstrated clearer responses. For instance, Diéguez et al. (13) observed higher acetate and total VFAs concentrations in piglets receiving EOs-based formulations. The numerical increases seen in our EOs-SH group may reflect a shift toward enhanced fermentative output.

As growth performance data from the same experimental system have been previously published, the physiological and intestinal findings reported here provide mechanistic support for the productivity improvements described in our previous study (23) in post-weaning piglets supplemented with a prebiotic formulation, EOs of oregano and clove plus SH and a combination of prebiotics and EOs. In that large-scale study (two replicates of 400 animals each), the group supplemented with EOs of oregano and clove plus SH achieved the highest average daily gain (0.46 ± 0.08 kg/day per animal; *p* < 0.001) and the lowest feed conversion ratio (1.75 ± 0.29 kg feed/kg weight gain; *p* < 0.001), as well as the highest body weight at day 70 (28.99 ± 4.20 kg; *p* < 0.001). At day 40, both the EOs-SH and the prebiotic groups showed similarly higher body weights compared with the control, with the prebiotic group presenting the numerically highest value (11.05 ± 1.56 kg), although no significant differences were detected between these two treatments. The combined treatment did not differ from the control at any time point. That work lends external validity to our observations of enterocyte preservation, enzymatic enhancement, and microbial modulation in the EOs-SH group. The fact that scFOSs alone improved certain functional markers, but that the combined treatment did not surpass the individual additives, mirrors the productivity results and supports the hypothesis of functional interference in combined supplementation. Taken together, these observations suggest a coherent mechanistic pathway: supplementation with EOs-SH enhances intestinal function and microbiota balance, which in turn may translate into improved growth performance, as previously evidenced in Mozo et al. (23).

In summary, the present study shows that scFOSs and the EOs-SH formulation exerted distinct and biologically meaningful effects on intestinal maturation, mucosal immunity, and microbial ecology during the early post-weaning period, whereas their combination did not provide additive benefits. The capacity of EOs-SH to preserve enterocyte functional mass, enhance brush-border activity, and modulate potentially proinflammatory taxa, together with the prebiotic-driven expansion of *Lactobacillus* and attenuation of mucosal immune activation in scFOSs-fed piglets, underscores their value as early nutritional interventions. Importantly, these findings align with a growing body of evidence supporting the prophylactic inclusion of functional feed additives as a means to stabilize gastrointestinal physiology and reduce vulnerability during the critical weaning window. In an era marked by increasing restrictions on antimicrobial use and the global imperative to curb antimicrobial resistance, such dietary strategies represent promising tools to enhance resilience in young pigs while reducing reliance on conventional antibiotics. Future studies with longer supplementation periods and integrative functional endpoints will help define the durability, scalability, and practical relevance of these interventions in animal production.

## Data Availability

The datasets presented in this study can be found in online repositories. The names of the repository/repositories and accession number(s) can be found in the article/Supplementary material.
